# Doctors’ and Nurses’ Social Media Ads Reduced Holiday Travel and COVID-19 infections: A cluster randomized controlled trial in 13 States

**Published:** 2021-06-21

**Authors:** Emily Breza, Fatima Cody Stanford, Marcela Alsan, Burak Alsan, Abhijit Banerjee, Arun G. Chandrasekhar, Sarah Eichmeyer, Traci Glushko, Paul Goldsmith-Pinkham, Kelly Holland, Emily Hoppe, Mohit Karnani, Sarah Liegl, Tristan Loisel, Lucy Ogbu-Nwobodo, Benjamin A. Olken, Carlos Torres, Pierre-Luc Vautrey, Erica Warner, Susan Wootton, Esther Duflo

**Affiliations:** ¶ Harvard University, Department of Economics, Cambridge, MA; † Harvard Kennedy School of Government, Cambridge, MA; # Online Care Group, Boston, MA; ‡ Massachusetts General Hospital, Department of Medicine- Neuroendocrine Unit, Department of Pediatrics- Endocrinology, Boston, MA; § Harvard Medical School, Boston, MA; || Massachusetts Institute of Technology, Department of Economics, Cambridge, MA; ** Stanford University, Department of Economics, Stanford, CA; *** Ludwig Maximilian University of Munich, Department of Economics, Munich, Germany; ## Bozeman Health Deaconess Hospital, Bozeman, MT; †† Yale University, New Haven, CT; ‡‡‡ Lynn Community Health Center, Lynn MA; §§ Johns Hopkins University, School of Nursing, Baltimore, MD; ||| St. Anthony North Family Medicine, Westminster, CO; ‡‡ Massachusetts General Hospital, Department of Psychiatry, Boston, MA; §§§ Massachusetts General Hospital for Children, Department of Pediatrics- General Pediatrics, Boston, MA; ¶¶ McLean Hospital, Department of Psychiatry, Belmont, MA; ††† Paris School of Economics, Paris, France; ¶¶¶ McGovern Medical School at The University of Texas Health Science Center at Houston, Houston, TX

## Abstract

During the COVID-19 epidemic, many health professionals started using mass communication on social media to relay critical information and persuade individuals to adopt preventative health behaviors. Our group of clinicians and nurses developed and recorded short video messages to encourage viewers to stay home for the Thanksgiving and Christmas Holidays. We then conducted a two-stage clustered randomized controlled trial in 820 counties (covering 13 States) in the United States of a large-scale Facebook ad campaign disseminating these messages. In the first level of randomization, we randomly divided the counties into two groups: high intensity and low intensity. In the second level, we randomly assigned zip codes to either treatment or control such that 75% of zip codes in high intensity counties received the treatment, while 25% of zip codes in low intensity counties received the treatment. In each treated zip code, we sent the ad to as many Facebook subscribers as possible (11,954,109 users received at least one ad at Thanksgiving and 23,302,290 users received at least one ad at Christmas). The first primary outcome was aggregate holiday travel, measured using mobile phone location data, available at the county level: we find that average distance travelled in high-intensity counties decreased by −0.993 percentage points (95% CI −1.616, −0.371, *p*-value 0.002) the three days before each holiday. The second primary outcome was COVID-19 infection at the zip-code level: COVID-19 infections recorded in the two-week period starting five days post-holiday declined by 3.5 percent (adjusted 95% CI [−6.2 percent, −0.7 percent], *p*-value 0.013) in intervention zip codes compared to control zip codes.

## One sentence summary

In a large scale clustered randomized controlled trial, short messages recorded by health professionals before the winter holidays in the United States and sent as ads to social media users led to a significant reduction in holiday travel, and to a decrease in subsequent COVID-19 infection at the population level.

Nurses and physicians are among the most trusted experts in the United States (1,2,3). Beyond the individual relationship with their patients, can these health professionals influence behavior at scale by spreading public health messages using social media?

During the COVID-19 crisis many healthcare professionals used social media to spread public health messages (3). For example, the Kaiser Family Foundation has sponsored a large project where doctors have recorded video to provide explanation about COVID-19 vaccination and dispel doubts (1). Since individual adoption of preventative behavior, from mask wearing and staying at home to vaccination, is key to the control of this and future pandemics, it is very important to know whether this communication is effective.

In previous work, we have shown, in online experiments, that video messages, recorded by a diverse group of doctors, affect the knowledge and behaviors of individuals and, and that these effects seem to be strong regardless of race, education, or political leanings (4,5). But there is no systematic evaluation of similar messages when distributed as part of large-scale public health campaigns. Furthermore, given the large sample required, it has not been possible so far to test the impact of such public health campaigns on COVID-19 infection, so the clinical significance of those finding was unclear.

In this study, we sought to estimate whether short video messages recorded by nurses and doctors, and sent on a massive scale as part of a social media ad campaign could impact both behavior and COVID-19 infections at the population level.

In November 2020, the number of COVID-19 cases was rapidly increasing in the United States. Due to concerns that holiday travel would lead to a surge in the epidemic, the Centers for Disease Control and Prevention (CDC) recommended that people stay home for the holidays.

In this context, we ran two large clustered randomized controlled trials with Facebook users. Before Thanksgiving and Christmas, physicians and nurses (all co-authors of this project) recorded twenty-second videos on their smart phones to encourage viewers to stay home for the holidays. Facebook subscribers in randomly selected zip codes in 820 counties in 13 states received these videos as sponsored content (ads). Over 11 million people received at least one ad before Thanksgiving (35% of users in the targeted regions), and over 23 million did before Christmas (66% of users in targeted regions).

The purpose of this study was to identify whether these short videos would influence population level holiday travel in the targeted regions, and in turn a decline in COVID-19 cases after the holidays.

## METHODS

### Trial Oversight

The design was approved by the institutional review board of the Massachusetts Institute of Technology (MIT) with Massachusetts General Hospital (MGH), Yale and Harvard ceding authority to MIT IRB. Messages were produced by the research team and approved to run (without modification) after going through Facebook’s internal policy review to ensure compliance with policies. Primary outcomes were registered on ClinicalTrials.gov. There was just one deviation from the pre-registration: we initially planned to construct the mobility outcome from fine-grained data. Since the publicly available mobility data is at the county level, we use county-level mobility data instead.

### Intervention

Messages encouraging viewers to stay home for the holidays were recorded on smartphones by six physicians before Thanksgiving, and nine physicians and nurses before Christmas who varied in age, gender, race and ethnicity.

For Thanksgiving, the script of the video was:

“This Thanksgiving, the best way to show your love is to stay home. If you do visit, wear a mask at all times. I’m [Title/ NAME] from [INSTITUTION], and I’m urging you: don’t risk spreading COVID. Stay safe, stay home.”

A similar script was recorded at Christmas. The videos were then disseminated as sponsored content to Facebook users from a page created for the project. The videos and the Facebook page are available on the project website (https://www.povertyactionlab.org/project/covid19psa). In the Supplementary Appendix, we provide details on the campaign and full scripts.

### Trial Design, Eligibility, Randomization and Recruitment

**Figure 1.**
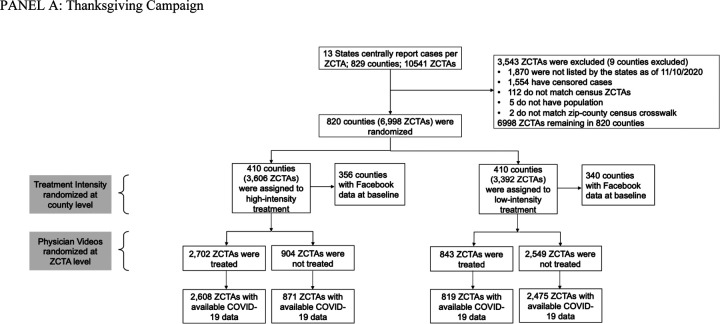

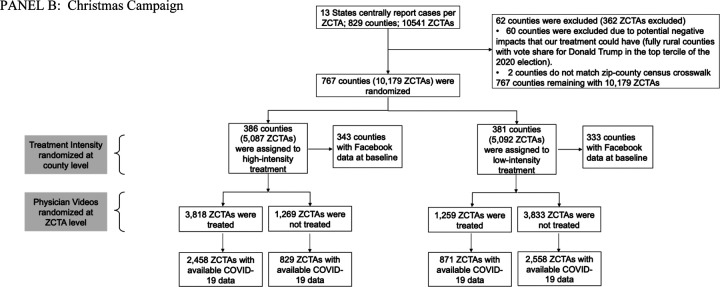
Consort Diagram

Eligibility for the trial and randomization strategy were determined by data availability and power considerations. Movement range data computed by Facebook is publicly available at the county-level. COVID-19 level data is available at the zip code level in some states. We thus randomized both at county and zip code level to have experimental variation for each level. The CONSORT diagram (Figure 1) describes the factorial design and the allocation of clusters to each arm.

Before the Thanksgiving campaign, we selected 13 states where weekly COVID-19 case-counts data were available at the zip code level (see maps in Figure S1a and S1b) and selected counties within these states where this data was available.

The research team randomly allocated counties to be “high-intensity” (H) or “low-intensity” (L) with probability ½ each. In H counties, the research team randomized zip codes into intervention with probability ¾ and control with probability ¼. In L counties, zip codes were randomized into intervention with probability ¼ and control with probability ¾. Randomization was performed with Stata prior to each of the two interventions.

The lists of zip codes for each intervention were then provided to our marketing partner AdGlow, who managed the advertising campaigns on Facebook. Within the treated zip codes, AdGlow ran ads to allocate the sponsored video content to users, aiming to reach the largest number of people given the advertising budget (see Supplement 1, Section A for further details about Facebook ad campaigns). The video messages were pushed directly into users’ Facebook feeds (three to five times per user on average), and users were then free to either watch, share, react to, or entirely ignore the content. We did not recruit individuals for the study and do not use individual level data. At Thanksgiving, 30,780,409 videos were pushed to 11,954,109 users, and at Christmas, 80,773,006 videos were sent to 23,302,290 users. AdGlow provided us with overall engagement figures: Each time a user had an opportunity to view a campaign message, 12.3% watched at least 3 seconds of the video at Thanksgiving and 12.9% at Christmas, while 1.7% watched at least 15 seconds at Thanksgiving and 1.4% at Christmas. Our engagement rates of 12–13% (measured as the total of clicks, 3-second views, shares, likes, and comments divided by total impressions) were well above industry standard benchmarks for Facebook ads, 1%−2%, and Facebook video posts, 6% (14, 15).

We determined that a sample of 820 counties would provide 80% power to detect effect sizes of 0.2 standard deviations for county-level outcomes, comparing intervention (H) vs. control (L). For outcomes with zip code level data, using intra-class correlations of 0.2 (0.475) a sample of 6,998 zip codes would provide 80% power to detect effect sizes of 0.057 (0.072) standard deviations.

### Outcomes

Our primary outcomes are county level mobility and zip code level COVID-19 infections reported to state health authorities, which we regularly retrieved from state websites beginning on November 12, 2020 (a list of the websites is provided in Supplement 1, Section B).

The movement range data are produced by aggregating location information obtained from mobile devices of Facebook users that opted to share their precise information with Facebook, and adding some noise for privacy protection (6,7) (see Supplement 1, Section B for further details). The *change in movement metric* is the percentage change in distance covered in a day compared to the same day of the week in the benchmark period of February 2–29, 2020. The mobility data describes the behavior throughout the day, for people who were in each county *that morning*. Since the campaign was targeted based on home location, we can only capture its impact on travel *away* from home, not back home. Thus, we define holiday travel as travel during the three days preceding each holiday. The *stay put metric* is the share of people who stay within a small geographical area (a “bing tile” of 600m*600m) in which they started the day. We used it to compute the *leave home* variable as = 1-*stay put* on the day of the holiday (Thanksgiving Day, Christmas Eve, and Christmas Day).

The second primary outcome we study is the number of new COVID-19 cases per fortnight, calculated from the cumulative case counts we manually retrieved from county or state webpages, one or twice a week and cleaned. Our primary outcome is the number of new COVID-19 cases detected in each zip code during fortnight that starts five days after each holiday: given the incubation period of five days, this is the one two-week period where we should see an impact.

### Statistical Analysis

The analysis was performed by original assigned group (intention to treat).

#### • Effect on Mobility (County-level)

At the county level, the analysis compares the “high-intensity” counties to the “low-intensity” counties. Because, on average, only 75% of the zip codes in high-intensity counties received the intervention, and 25% in low-intensity counties received the intervention, this is “an intention to treat” specification which is a lower bound of the effect of treatment.

For any day or set of days, the coefficient of interest is *β*_1_ in the OLS regression:

(1) $$y_{it}=\beta_{0}+\beta_{1}\text{Hig}h_{i}+\beta_{2}y_{i0}+{\text{X}}_{\text{i}}\beta_{3}+\varepsilon it$$

where *y*_*it*_ is the outcome of interest on day t, and *y*_*i*0_ its baseline value. This regression is estimated for both campaigns together, and for each separately. Standard errors are adjusted for heteroskedasticity, and clustering at zip code levels when both campaigns are pooled (we also provide randomization inference p-values) (8). We present a regression controlling for state fixed effects and a set of county level outcomes chosen via machine learning (9) in Table S4 (in supplementary appendix).

#### • Effect on Number of COVID-19 Cases (Zip Code-level)

To measure the effect on COVID-19 cases reported in each zip code, we run the regression:

(2), $$\text{Asinh}\left(\text{fortnightly COVI}D_{it}\right)=\beta_{0}+\beta_{1}\text{Treate}d_{i}+\beta_{2}\text{ log}\left(\text{cumulative COVI}D_{i0}\right)+\beta_{3}^{T}\text{stratu}{\text{m}}_{\text{i}}+\varepsilon it$$

Where *fortnightly COVID*_*it*_ is the number of new cases of COVID-19 detected in the fortnight beginning five days after each holiday (for primary outcome results), is a dummy that indicates that zip code *i* was a treated zip code. The hyperbolic sine transformation is appropriate when the data is approximately lognormal for higher values, but a small number of observations have zero cases (10,11) (also see Supplement 1, Section C). The coefficient of “Treated” can be interpreted as a proportional change. In the supplementary appendix we explore robustness to other commonly used ways to handle zeros. We also investigate robustness by estimating the same regression for other fortnights.

Regression (2) is estimated for both campaigns pooled, and for the Thanksgiving campaign and the Christmas campaign separately, with county fixed effects (the randomization strata). Standard errors adjust for heteroskedasticity (and clustering for the pooled specification) and we compute p-values with randomization inference. We estimate the impact of treatment overall, and separately in the two strata (high- and low-intensity counties).

In supplementary material, we also explore heterogeneity of effects by prior COVID-19 circulation and demographic variables. Analyses were performed using R, version 4.0.3, including the following packages (versions): *stats* (4.0.3), *tidyverse* (1.3.0), *estimatr* (0.28.0), *readr* (1.4.0), *dplyr* (1.0.5), *lubridate* (1.7.10), *hdm* (0.3.1), *car* (3.0.10), *MASS* (7.3.53), sandwich (3.0.0), foreign (0.8.80), readstata13 (0.9.2), readxl (1.3.1), quantreg (5.75). The data and all the statistical codes will be made available upon publication.

### Role of the Funding Source

Facebook provided the ad credits used to show the ads and connected the research team with AdGlow, the marketing partner. The ad content went through the usual internal policy review at Facebook for compliance with policies. Facebook had no other role in the design or conduct of the trial, and no role in the interpretation of the data or preparation of the manuscript.

## RESULTS

### Trial Population

Of the 8,671 potentially eligible zip codes in the 13 states in the studies, 1,554 were removed before the Thanksgiving campaign because of missing COVID-19 infection data, and 119 were removed because they could not be matched to county-level census data, yielding a sample of 6998 zip codes in 820 counties. Prior to the Christmas campaign, 60 fully rural counties in the top tercile of votes for Donald Trump in the 2020 election were removed from the study. This was done out of caution and to avoid adverse effects. The research team was concerned that the messaging campaign might have adverse unintended effects in very rural, heavily Republican-leaning counties given the growing polarization in December. The remaining sample had 767 counties. We included in the campaign all zip codes in the intervention in the selected counties (even if they could not be matched to COVID-19 infection data). For the COVID-19 outcomes, we have a final sample of 6716 zip codes. The realized sample size of 820 counties at Thanksgiving and 767 counties at Christmas was close enough to the original sample size to not lead to significant loss in power.

Summary statistics on the sample that was randomized are shown in Table 1 (Figures S1a and S1b in the supplementary appendix shows their localization on the map). Counties had on average 36% Democrats, 62% Republicans (based on election share in 2020) and 46% of zip codes were classified as urban. On November 13, 2020, distance travelled was 8.73% lower than during the benchmark month of February 2020; In the Christmas sample, it was 8.89% lower. In both samples, 82.4% of people left home on November 13, 2020.

### Effects of the Campaign on the Mobility of Facebook users

**Figure 2.**
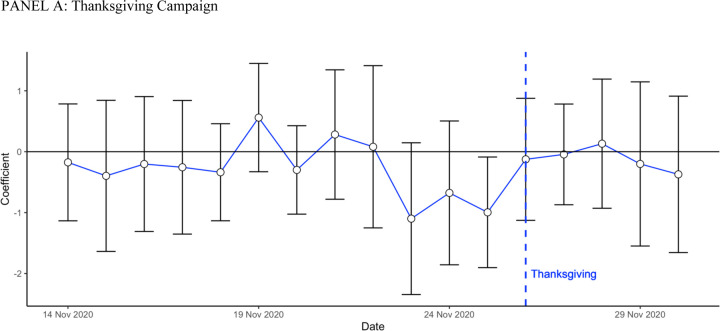

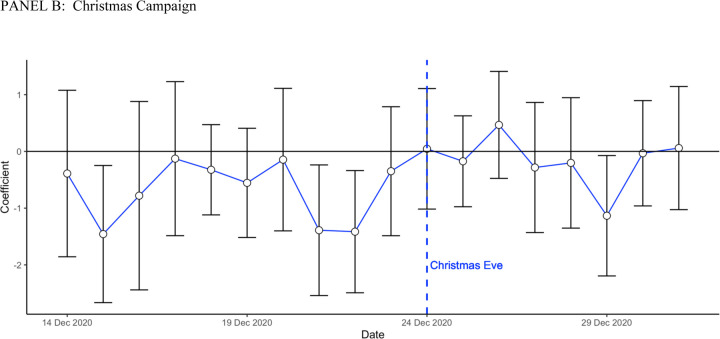
Day-by-day Difference between High and Low Intensity Counties on Distance Traveled relative to February 2020* * These figures display a day by day estimation of the regression equation (1). The outcome is the distance traveled relative to February 2020.

Figure 2 shows day-by-day regressions estimates of equation (1). Distance travelled away from the morning location declined a few days before each holiday in high-intensity counties, relative to low-intensity counties.

Table 2 shows that, pooling both campaigns together, distance travelled three days before each holiday was 4.384 percent lower than in February 2020 in high-intensity counties, and 3.597 percent lower in low-intensity counties. The adjusted difference was 0.993 percentage points (95% CI −1.616, −0.371, p. value 0.002). The effects were very similar at Thanksgiving (adjusted difference: −0.924 percentage point, 95% CI (−1.785, −0.063, p. value 0.035) and Christmas (adjusted difference: −1.041 percentage point 95% CI −1.847, −0.235, p value 0.011).

The intervention had no impact on the share of people leaving home on the day of the holiday (Table 2 and supplementary appendix Figure S2). On average, 72.33% of people left their home tile on the day of the holiday in high-intensity counties, and 72.39% in low-intensity counties (adjusted difference 0.030 95% CI (−0.361, 0.420), p. value 0.881).

Table S4 in the supplement shows that these results are robust to adding control variables chosen by machine learning from a large set of county-level covariates (12).

### Effect of the Campaign on COVID-19 Cases

Table 3 shows that the campaigns were followed by a drop in COVID-19 cases in treated zip codes, relative to control zip codes, for the two-week period beginning five days after the holiday. The adjusted difference in asinh (covid) was 0.035 (adjusted 95% CI [−0.062, −0.007], p. value 0.013), which can be interpreted as a 3.5% reduction in COVID-19 cases. The effects were slightly smaller in magnitude at Thanksgiving (adjusted difference: −0.027 (adjusted 95% CI [−0.059, +0.005], p. value 0.097) than at Christmas (adjusted difference, −0.042 95% CI [−0.073, −0.012] p. value 0.007). These results are robust to alternative ways to treat zero (Tables S6a, S6b, and S6c in the supplement).

**Figure 3.**
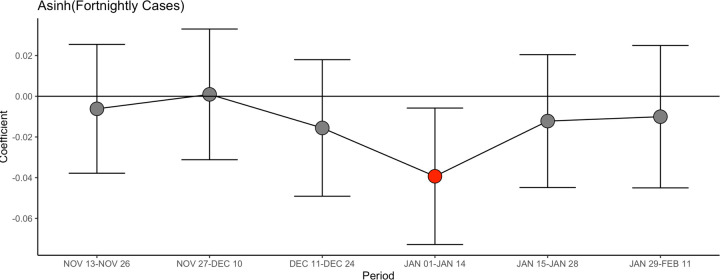
Difference between treated and control zip codes (Christmas intervention), for various periods* * Each dot represents the point estimate of estimating equation (2) for the given period. The whiskers are the 95% confidence intervals

To provide evidence that these differences are indeed due to the campaign, and not to any pre-existing difference, Figure 3 show the results of estimating equation (2) for a number 2-weeks periods (omitting the five days following Christmas). There is no significant difference in intervention and comparison zip codes in any period other than the period where we expected an impact. This makes it very unlikely that the difference in COVID-19 cases is due to random chance.

### Treatment Effect Heterogeneity

We test for several dimensions of heterogeneity of the effect of the campaign on mobility and COVID-19 infection in Tables S2a–b and S3a–g in the supplementary appendix: baseline COVID-19 infection, urban versus rural counties, education, and majority Republican versus majority Democratic counties.

We found no significant difference in the impact of the campaign either on mobility or COVID-19 cases by level of education, or between Republican and Democratic counties, or between rural and urban counties. We also did not find that the interaction between political leaning and urban designation is significant (Tables S3e and S3f in the supplement). The effects on COVID-19 infections are lower in counties with high infection at baseline.

## DISCUSSION

There was widespread concern before the Thanksgiving and Christmas holidays that heavy travel and mixing households would lead to an increase in COVID-19 patients. Indeed, households did travel more around the holidays, though even then mobility remained lower than its February 2020 level.

In counties where a larger proportion of zip codes were randomly assigned to a high-coverage Facebook ad campaigns in which clinicians encouraged people to stay home before the Thanksgiving and Christmas holidays, Facebook users reduced the distance they travelled in the three days before the holidays. Although they were less likely to leave their homes on the day of the holiday, the clinical importance of this latter finding is unclear, since they could either have been spending time outside or visiting other households.

A potential concern before the campaign was that in a polarized environment, a campaign such as this one could be effective in some communities and backfire in others (this is why we excluded a few counties in the Christmas campaign). But the effects did not seem to depend on county characteristics, including political leanings. These findings accord with previous research that found that individuals are responsive to physician delivered messages, regardless of political affiliation (5).

We found a significant impact on new COVID-19 infections reported by health authorities 5 to 19 days later. These effects might be under-estimated, because the treatment and control zip codes are very close to each other, and the reductions in infection in treatment zip codes might also have led to a decrease in infection in neighboring places.

There are several limitations of the study. First, it is was conducted with Facebook subscribers and mobility is collected for Facebook users. Although Facebook has a remarkable reach, this remains just one type of media. Second, it was an ad campaign. The messages might have been more effective if they had been relayed by celebrities or locally known figures (12,13). Third, we tested one kind of message, recorded by clinicians on smartphones. The results could be different changing message content, identity of the messenger, length of message, production value of the videos, or name recognition of the originating organization.

Despite these limitations, the findings provide evidence that clinicians can be an effective channel to communicate life-saving information at scale, through social media. This a new role that physicians and nurses embraced during the COVID-19 crisis, and we demonstrate that this is another way in which they can prevent illness and save lives.

These findings also demonstrate, in a clustered randomized control trial, the impact of a travel reduction, a key non-clinical intervention whose impact had not been evaluated in a randomized controlled trial before.

The findings suggest directions for future work. In particular, would similar messages be effective in encouraging COVID-19 vaccine uptake?

## Supplementary Material

1

## Figures and Tables

**Table 1.** Summary Statistics*

	Thanksgiving sample		
	Sample	High Intensity counties	Low Intensity counties
**Number of counties**	820	410	410
**Movement, mean (sd)**			
Baseline Movement Metric	−8.73 (6.77)	−8.58 (7.10)	−8.88 (6.42)
Baseline Leave Home	82.41 (2.47)	82.33 (2.42)	82.49 (2.53)
Missing Baseline Facebook outcomes	0.15 (0.36)	0.13 (0.34)	0.17 (0.38)
**Covid-19, mean (sd)**			
Baseline Fortnightly Cases	590.30 (2297.94)	683.90 (3032.94)	496.70 (1165.17)
Baseline Fortnightly Deaths	5.07 (17.63)	5.51 (22.35)	4.64 (11.08)
**Demographic, mean (sd)**			
Share Urban	0.46 (0.34)	0.47 (0.34)	0.44 (0.34)
Share Democrats	0.36 (0.15)	0.36 (0.15)	0.35 (0.15)
Share Republicans	0.62 (0.15)	0.62 (0.16)	0.63 (0.15)
Population in 2019	112654 (317672)	122491 (349501)	102818 (282369)

	Christmas sample		
	Sample	High Intensity counties	Low Intensity counties
**Number of counties**	767	386	381
**Movement, mean (sd)**			
Baseline Movement Metric	−8.89 (6.72)	−8.69 (6.88)	−9.09 (6.56)
Baseline Leave Home	82.42 (2.41)	82.40 (2.43)	82.44 (2.40)
Missing Baseline Facebook outcomes	0.12 (0.32)	0.11 (0.32)	0.13 (0.33)
**Covid-19, mean (sd)**			
Baseline Fortnightly Cases	626.84 (2371.71)	654.77 (3067.53)	598.54 (1343.02)
Baseline Fortnightly Deaths	5.38 (18.19)	5.70 (23.07)	5.07 (11.29)
**Demographic, mean (sd)**			
Share Urban	0.49 (0.33)	0.48 (0.33)	0.50 (0.33)
Share Democrats	0.37 (0.15)	0.37 (0.15)	0.37 (0.15)
Share Republicans	0.61 (0.15)	0.61 (0.15)	0.61 (0.15)
Population in 2019	119811 (327266)	116787 (344511)	122875 (309239)

* These tables presents summary statistics on baseline variables, for both Thanksgiving and Christmas samples. Baseline = Nov 13.

**Table 2.** Effect of Treatment on Movement Outcomes*

			Mean (95% CI)		OLS model			Number of days*counties
Campaign	Outcome	Period	High county	Low county	High county (95% CI)	p-value	RI p-value	
**Both campaigns**	**Distance Traveled**	**from d-3 to d-1**	−4.384 (−4.973,−3.796)	−3.603 (−4.254,−2.952)	−0.993 (−1.616,−0.371)	0.002	0.002	4059
	**Share Ever Left Home**	**Thanksgiving (Nov 26) or Christmas (Dec 2425)**	72.326 (72.012,72.639)	72.381 (72.092,72.670)	0.030 (−0.361,0.420)	0.881	0.879	2017
**Thanksgiving**	**Distance Traveled**	**from d-3 to d-1**	−6.082 (−6.822,−5.341)	−5.320 (−6.113,−4.527)	−0.924 (−1.785,−0.063)	0.035	0.030	2072
	**Share Ever Left Home**	**Thanksgiving (Nov 26)**	71.308 (70.885,71.731)	71.468 (71.071,71.866)	0.012 (−0.438,0.461)	0.959	0.966	689
**Christmas**	**Distance Traveled**	**from d-3 to d-1**	−2.603 (−3.279,−1.927)	−1.823 (−2.588,−1.057)	−1.041 (−1.847,−0.235)	0.011	0.012	1987
	**Share Ever Left Home**	**Christmas (Dec 24–25)**	72.859 (72.507,73.210)	72.852 (72.520,73.185)	0.095 (−0.289,0.479)	0.629	0.661	1328

* This table provides the control and treatment means at the county level and different periods, in addition to the estimate of the treatment coefficient in equation (1). Standard errors are clustered at the county level. 95% CI are reported in parentheses.

**Table 3.** Treatment Effect on COVID-19 Cases at Zip Code Level*

				Mean (CI 95%)		OLS model			Num
Campaign	Outcome	Period	County treatment	Treatment	Control	Treatment (CI 95%)	p-value	RI p-value	
**Both campaigns**	**Asinh(Fortnightly Cases)**	**Dec/Jan 1–14**	**All**	4.350 (4.302,4.398)	4.370 (4.323,4.417)	−0.035 (−0.062,−0.007)	0.013	0.009	
			**Low Intensity**	4.359 (4.273,4.445)	4.358 (4.305,4.411)	−0.032 (−0.067,0.004)	0.080	0.097	
			**High Intensity**	4.347 (4.295,4.399)	4.407 (4.325,4.489)	−0.039 (−0.075,−0.003)	0.033	0.038	
**Thanksgiving**	**Asinh(Fortnightly Cases)**	**Dec 1–14**	**All**	4.333 (4.278,4.388)	4.298 (4.243,4.353)	−0.027 (−0.059,0.005)	0.097	0.108	
			**Low Intensity**	4.284 (4.170,4.399)	4.256 (4.192,4.320)	−0.015 (−0.063,0.033)	0.535	0.498	
			**High Intensity**	4.348 (4.285,4.411)	4.418 (4.313,4.523)	−0.039 (−0.082,0.004)	0.078	0.096	
**Christmas**	**Asinh(Fortnightly Cases)**	**Jan 1–14**	**All**	4.368 (4.310,4.425)	4.442 (4.385,4.499)	−0.042 (−0.073,−0.012)	0.007	0.010	
			**Low Intensity**	4.429 (4.312,4.547)	4.456 (4.391,4.522)	−0.048 (−0.091,−0.006)	0.025	0.043	
			**High Intensity**	4.346 (4.280,4.412)	4.396 (4.281,4.510)	−0.036 (−0.080,0.008)	0.108	0.111	

* This table provides the control and treatment means at the zip code level, in addition to the estimate of the treatment coefficient in equation (2). The outcome is the inverse hyperbolic sine of the fortnightly cases, during a period which starts five to seven days after the event (Thanksgiving or Christmas). 95% CI are reported in parentheses. Standard errors are clustered at the zip level.

## References

[1] Altman D. Why Doctors and Nurses Can Be Vital Vaccine Messengers. Kaiser Family Foundation, 2021. Available from: https://www.kff.org/coronavirus-covid-19/perspective/why-doctors-and-nurses-can-be-vital-vaccine-messengers/

[2] Hamel L, Kirzinger A, Lopes L, Kearney A, Sparks G, and Brodie M. KFF COVID-19 Vaccine Monitor: January 2021. Kaiser Family Foundation, 2021. Available from: https://www.kff.org/report-section/kff-covid-19-vaccine-monitor-january-2021-vaccine-hesitancy/

[3] McDonnell Nieto del Rio G. Doctors plead with Americans to take the virus surge seriously. The New York Times, 2020. Available from: https://www.nytimes.com/live/2020/11/15/world/covid-19-coronavirus#doctors-plead-with-americans-to-take-the-virus-surge-seriously.

[4] Alsan M, Cody Stanford F, Banerjee A, Comparison of Knowledge and Information-Seeking Behavior After General COVID-19 Public Health Messages and Messages Tailored for Black and Latinx Communities: A Randomized Controlled Trial. Ann Intern Med. 2021;174(4):484–492. 33347320 10.7326/M20-6141 PMC7774591

[5] Torres C, Ogbu-Nwobodo L, Alsan M, Effect of Physician-delivered COVID-19 Public Health Messages and Messages Acknowledging Racial Inequity on Black and White Adults’ Knowledge, Beliefs, and Practices Related to COVID-19: A Randomized Clinical Trial. Forthcoming in JAMA network open, July 15, 2021 10.1001/jamanetworkopen.2021.17115 PMC8280971 34259846

[6] Maas P, Iyer S, Gros A, 2020. “Facebook Disaster Maps: Aggregate Insights for Crisis Response and Recovery.” KDD 2019;19:3173.

[7] Movement Range Maps. Facebook Data for Good, 2021. Available from: https://data.humdata.org/dataset/movement-range-maps

[8] Imbens GW, and Rubin DB. Causal inference in statistics, social, and biomedical sciences. Cambridge, England: Cambridge University Press, 2015:1–625.

[9] Chernozhukov V, Chetverikov D, Demirer M, Double/debiased machine learning for treatment and structural parameters. Econom J 2018;21(1):C1–C68.

[10] Burbidge JB, Magee L, Robb AL. Alternative Transformations to Handle Extreme Values of the Dependent Variable. J Am Stat Assoc 1988; 83(401):123–127.

[11] Zhang M, Fortney JC, Tilford JM, Rost KM. An Application of the Inverse Hyperbolic Sine Transformation—A Note. Health Serv Outcomes Res Methodol 2000; 1:165–171.

[12] Alatas V, Chandrasekhar AG, Mobius M, Olken BA, Paladines C. When celebrities speak: A nationwide Twitter experiment promoting vaccination in Indonesia. No. W25589. National Bureau of Economic Research, 2019.

[13] Banerjee A, Alsan M, Breza E, . Messages on Covid-19 prevention in India increased symptoms reporting and adherence to preventive behaviors among 25 million recipients with similar effects on non-recipient members of their communities. No. W27496. National Bureau of Economic Research, 2020.

[14] What’s a Good Facebook Engagement Rate? Amplify Partners, 2020. Available from: https://acumen.aamplify.partners/whats-a-good-facebook-engagement-rate

[15] Digital 2020 April Global Statshot Report. We Are Social, 2020. Available from: https://datareportal.com/reports/digital-2020-april-global-statshot

