# Supplementary material for: Doctors’ and Nurses’ Social Media Ads Reduced Holiday Travel and COVID-19 infections: A cluster randomized controlled trial in 13 States
Source: ArXiv. 2021 Jun 21:arXiv:2106.11012v1. Preprint. [Version 1] (PMC8219102)
Supplement: 1 [file NIHPP2106.11012V1-supplement-1.pdf]

Emily Breza, Ph.D.
Harvard Department of Economics
1805 Cambridge Street
Cambridge, MA 02138

Marcella Alsan, M.D. M.P.H. Ph.D.
Harvard Kennedy School
79 John F. Kennedy Street
Cambridge, MA 02138

Burak Alsan, M.D.
Online Care Group
75 State Street
Boston, MA 02109

Abhijit Banerjee, Ph.D.
MIT Department of Economics
77 Massachusetts Avenue
Cambridge, MA 02139

Fatima Cody Stanford, M.D. M.P.P.
MGH Weight Center
50 Staniford Street, Suite 430
Boston, MA 02114

Arun G. Chandrasekhar, Ph.D.
Stanford Department of Economics
579 Jane Stanford Way
Stanford, CA 94305-6072

Sarah Eichmeyer, Ph.D.
University of Munich
Center for Economic Studies (CES)
Schackstr. 4 / I
80539 Munich
Germany

Traci Glushko, M.S.
Bozeman Health Deaconess Hospital
915 Highland Boulevard
Bozeman, MT 59715

Paul Goldsmith-Pinkham, Ph.D.
Yale School of Management
165 Whitney Avenue
New Haven, CT 06511

Kelly Holland, M.D.
Lynn Community Health Center
269 Union Street
Lynn, MA 01901

Emily Hoppe, M.S.
Johns Hopkins School of Nursing
525 N. Wolfe Street
Baltimore, MD 21205

Mohit Karnani, M.Sc.
MIT Department of Economics
77 Massachusetts Avenue
Cambridge, MA 02139

Sarah Liegl, M.D.
St. Anthony North Family Medicine
2551 W 84th Ave
Westminster, Colorado 80031

Tristan Loisel, M.Sc.
Paris School of Economics
48 Boulevard Jourdan
75014 Paris, France

Lucy Ogbu-Nwobodo, M.D.
Massachusetts General Hospital
55 Fruit St
Boston MA 02114

Benjamin A. Olken, Ph.D.
MIT Department of Economics
77 Massachusetts Avenue
Cambridge, MA 02139

Carlos Torres, M.D.
Chelsea HealthCare Center
151 Everett Avenue
Chelsea, MA 02150

Pierre-Luc Vautrey, M.Sc.
MIT Department of Economics
77 Massachusetts Avenue
Cambridge, MA 02139

Erica Warner, Sc.D. M.P.H.
Massachusetts General Hospital
55 Fruit St
Boston, MA 02114

Susan Wootton, M.D.
University of Texas Health Science Center
7000 Fannin Street #1200
Houston, TX 77030

Esther Duflo, Ph.D.
MIT Department of Economics
77 Massachusetts Avenue
Cambridge, MA 02139

Supplementary Appendix

Table of Contents

Supplement 1. Methods, and Results

Methods

Section A. Facebook Ad Campaigns

Section B. Outcomes

Section C. Regression Models Details

Results

Section D. Figures and Tables

Figure S1a. Randomized counties (Thanksgiving campaign)

Figure S1b. Randomized counties (Christmas campaign)

Figure S2. Day by day difference between high and low intensity counties on Share Ever Left Home*

Table S2a. Analyses of Mobility Outcomes by Baseline Covid-19 Cases*

Table S2b. Analyses of Covid-19 Outcome by Baseline Covid-19 Cases *

Table S3a. Analyses of Mobility Outcomes by Party Majority*

Table S3b. Analyses of Covid Outcome by Party Majority*

Table S3c. Analyses of Mobility Outcomes: Urban vs Rural*

Table S3d. Analyses of Covid Outcome: Urban vs Rural *

Table S3e Analyses of Mobility Outcomes by Republican Majority x Urban Majority*

Table S3f. Analyses of Covid Outcome by Republican Majority x Urban Majority*

Table S3e. Analyses of Mobility Outcomes by Education*

Table S3g. Analyses of Covid Outcome by Education*

Table S4. Effect of Intervention on Movement Outcomes, with Double Post Lasso Control Variables*

Table S5. Effect of Intervention on Covid-19 Outcome: median regression*
Table S6a. Effect of Intervention on Covid-19 Outcome (both campaigns), robustness to function form
Table S6b. Effect of Intervention on Covid-19 Outcome (Thanksgiving campaign), robustness to functional form
Table S6c. Effect of Intervention on Covid-19 Outcome (Christmas campaign), robustness to functional form
Section E. References
Supplement 2. Statistical Analysis Plan

List of Investigators

Emily Breza, Ph.D.,[¶] Marcella Alsan, M.D. Ph.D.,[†,*] Burak Alsan, M.D.,[#] Abhijit Banerjee, Ph.D.,[‖] Fatima Cody Stanford, M.D. M.P.H, M.P.A.,M.B.A.,[‡,§,*] Arun G. Chandrasekhar, Ph.D.,[**] Sarah Eichmeyer, Ph.D., [***] Traci Glushko, M.S.,[##] Paul Goldsmith-Pinkham, Ph.D.,[††] Kelly Holland, M.D.,[‡‡‡] Emily Hoppe, M.S., [§§] Mohit Karnani, M.Sc. [‖], Sarah Liegl, M.D., [|||] Tristan Loisel, M.Sc. [†††], Lucy Ogbu-Nwobodo, M.D. M.S. M.A.S., [§,‡‡,¶¶] Benjamin A. Olken Ph.D.,[‖] Carlos Torres, M.D., [§,§§§] Pierre-Luc Vautrey, M.Sc. [‖], Erica Warner, Sc.D., M.P,H., ,[‡,§,*] Susan Wootton, M.D., [¶¶¶] Esther Duflo, Ph.D.[‖]

Affiliations:

[¶] Harvard University, Department of Economics, Cambridge, MA
[†] Harvard Kennedy School of Government, Cambridge, MA
[#] Online Care Group, Boston, MA
[‡] Massachusetts General Hospital, Department of Medicine- Neuroendocrine Unit, Department of Pediatrics- Endocrinology, Boston, MA
[§] Harvard Medical School, Boston, MA
[‖] Massachusetts Institute of Technology, Department of Economics, Cambridge, MA
[**] Stanford University, Department of Economics, Stanford, CA
[***] Ludwig Maximilian University of Munich, Department of Economics, Munich, Germany

[##] Bozeman Health Deaconess Hospital, Bozeman, MT
[††] Yale University, New Haven, CT
[‡‡‡] Lynn Community Health Center, Lynn MA
[§§] Johns Hopkins University, School of Nursing, Baltimore, MD
[‖] St. Anthony North Family Medicine, Westminster, CO
[‡‡] Massachusetts General Hospital, Department of Psychiatry, Boston, MA
[§§§] Massachusetts General Hospital for Children, Department of Pediatrics- General Pediatrics, Boston, MA
[¶¶] McLean Hospital, Department of Psychiatry, Belmont, MA
[†††] Paris School of Economics, Paris, France
[¶¶¶] McGovern Medical School at The University of Texas Health Science Center at Houston, Houston, TX

Supplement 1. Methods, and Results

Methods

Section A. Facebook Ad Campaigns

We disseminated the messages using a Facebook advertising campaign that was managed by AdGlow, our marketing partner. On the Facebook advertising platform, there are many ways to structure a campaign. We selected a "reach" objective, which attempts to maximize the number of Facebook users seeing the ads, along with the number of times each user sees the ad, over a daily horizon or the lifetime of the campaign given the campaign budget. The Thanksgiving campaign had a daily "reach" objective, while the Christmas campaign had a lifetime "reach" objective. Facebook uses an algorithm to implement the campaign objective. (More information is available at https://www.facebook.com/business/help/218841515201583?id=816009278750214.)

An important element of the algorithm is the Facebook Ads Auction. All active ad campaigns define a target audience. For both of our campaigns, the target audience consisted of all Facebook users in the specified zip-codes. Every time there is an opportunity to show an ad to a user, there may be many active campaigns targeting that type of individual. An auction is used to determine the cost of the ad and which ad is shown to the user at that time, and the auction winner is the advertiser with the highest total value. Total value is a combination of three factors: the bid of each advertiser; the estimated action rate (whether the user engages with the ad in the desired way); ad quality, which is measured by Facebook and reflects feedback from previous viewers and assessments of so-called "low-quality attributes." By defining total

value as more than simply the advertiser's bid, ads that are estimated to create more user engagement or that are of higher quality can beat ads with higher bids in the auction. In this way, the Facebook ad campaign algorithm and Ads Auction led to the delivery of campaign materials to 11,954,108 users at Thanksgiving and 23,302,290 users at Christmas. (More information about the Facebook Ads Auction is available at https://www.facebook.com/business/help/430291176997542?id=561906377587030.)

Section B. Outcomes

County level mobility data

Our mobility outcomes come from the publicly-available Facebook Movement Range dataset, which can be downloaded at https://data.humdata.org/dataset/movement-range-maps. The data are constructed from location information collected by Facebook from users who have opted into Location History sharing and are aggregated to the county level. The publicly released data is subjected to a differential privacy framework to maintain the privacy of individual Facebook users. First, regions with fewer than 300 users in a given data are omitted from the data set. Second, random noise is added during the construction of each metric to limit the risk of being able to identify individual users.

We use both the Change in Movement metric and the Stay Put metric in our analysis. Both are calculated daily and cover the period from 8pm to 7:59pm local time. Both metrics are based off of changes in locations across level-16 Bing tiles, which each represent an area of approximately 600m x 600m.

Change in Movement is a measure of how many tiles the average Facebook user starting in a given county travels through during the day. More specifically, the variable is constructed for each county, on each day following 5 steps: 1) the number of tiles visited is calculated for each user and is top-coded at 200; 2) the total number of tiles visited by all users in that county-day observation is calculated by summing over the top-coded tiles measure; 3) random noise is added to the total tiles measure following a LaPlace distribution with parameters selected to satisfy Facebook's differential privacy targets; 4) the noisy total tiles variable is scaled by Facebook users observed in the data to generate an average for that day in each county; 5) finally, the average movement measure is scaled by an average baseline measurement for the county taken on the same day of the week between February 2-29, 2020.

Stay Put is calculated as the fraction of observed users in a given county who do not leave a single level-16 Bing tile for the whole day. Specifically, in constructing the public version of this metric, 5 steps are followed: 1) a binary indicator is calculated for each user based on whether they remained in a single level-16 Bing tile for the entire day; 2) the total number of users in each county staying put is generated;

steps 3)-5) from the Change in Movement calculation are followed. When we use the Stay Put metric in our analysis, we instead create Leave Home = 1 - Stay Put so that larger values indicate more movement.

The Facebook Movement Range data are described in further detail at https://research.fb.com/blog/2020/06/protecting-privacy-in-facebook-mobility-data-during-the-covid-19-response/.

Zip Code level COVID-19 data

The COVID-19 data was retrieved twice a week from the following State health websites. The data is reported by hospital or labs to the centralized State wide health department, which publishes the data we collected and used. Most states report positive cases based on PCR tests, but some (AZ, IL, MN) combine confirmed with probable cases.

Different states have different formats to report their data: some had clean spreadsheets, others had spreadsheets that were reformatted, and others had pdfs, that had to be converted into spreadsheets and cleaned. The data was retrieved manually and organized.

States reported the cumulative cases reported in each zip code. Cases are assigned to a zip code based on the address of the person who tested positive.

Some zip codes were not listed on the states' websites. (we observe around 8k unique zips before dropping the censored ones, whereas the total zip count for these 13 states is a bit over 10k). There are multiple reasons for this, the most popular being aggregation of small zip codes into larger ones (there were other situations, like suppressing Tribal zips, or simply suppressing small zips instead of aggregating them), and the data were censored for zip codes with low case counts,

We cleaned and appended all the data we collected, totaling 6998 unique zip codes with unsuppressed, non-censored data.

A list of the website from which the data was retrieved appears here.

AZ: https://www.azdhs.gov/covid19/data/index.php

AR: https://achi.net/covid19/

FL: https://experience.arcgis.com/experience/96dd742462124fa0b38ddedb9b25e429

IL: https://www.dph.illinois.gov/covid19/covid19-statistics

IN: https://hub.mph.in.gov/dataset?q=COVID

ME: https://www.maine.gov/dhhs/mecdc/infectious-disease/epi/airborne/coronavirus/data.shtml

MD: https://coronavirus.maryland.gov/datasets/mdcovid19-master-zip-code-cases/data

MN: https://www.health.state.mn.us/diseases/coronavirus/stats/index.html

NC: https://covid19.ncdhhs.gov/dashboard

OK: https://looker-dashboards.ok.gov/embed/dashboards/80

OR: https://govstatus.egov.com/OR-OHA-COVID-19

RI: https://ri-department-of-health-covid-19-data-rihealth.hub.arcgis.com/

VA: https://www.vdh.virginia.gov/coronavirus/covid-19-data-insights/

Section C. Regression Models Details

Inverse Hyperbolic Sine function:

The hyperbolic sine function is given by: $sinh(x) = \frac{e^x - e^{-x}}{2}$, and the inverse hyperbolic sine function, is given by $asinh(x) = ln(x + \sqrt{x^2 + 1})$.

We chose to transform the fortnightly cases with this function, because it has the property to be equivalent to x close to 0, and to be equivalent to $ln(x)$ when $x \to +\infty$: $asinh(x) \underset{x \to 0^+}{\sim} x$, $asinh(x) \underset{x \to +\infty}{\sim} ln(x)$. It behaves like a logarithm for most our our observations, except that there is no singularity at 0.

Results

Section D. Figures and Tables

Figure S1a. Randomized counties (Thanksgiving campaign)

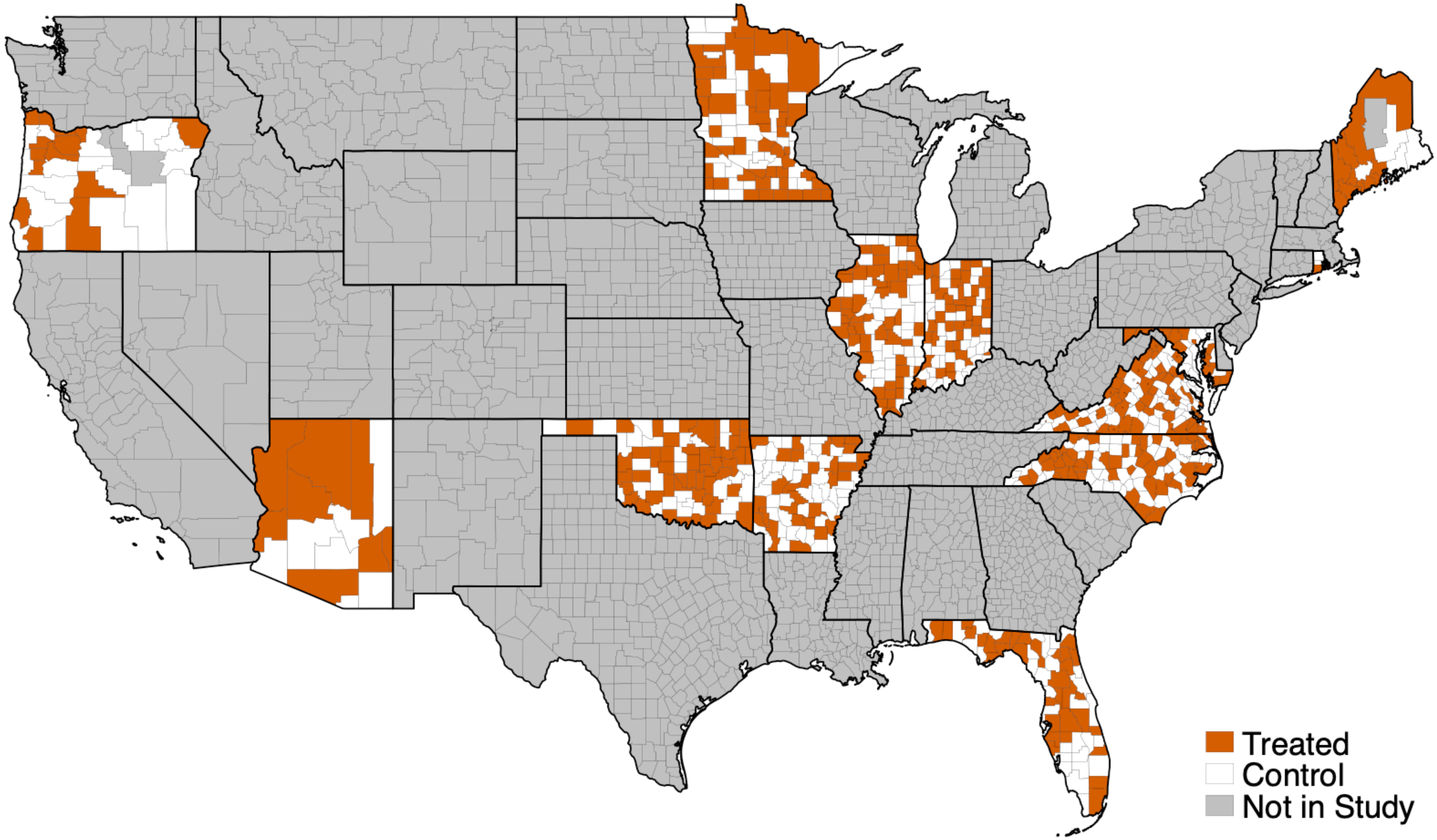

Figure S1b. Randomized counties (Christmas campaign)

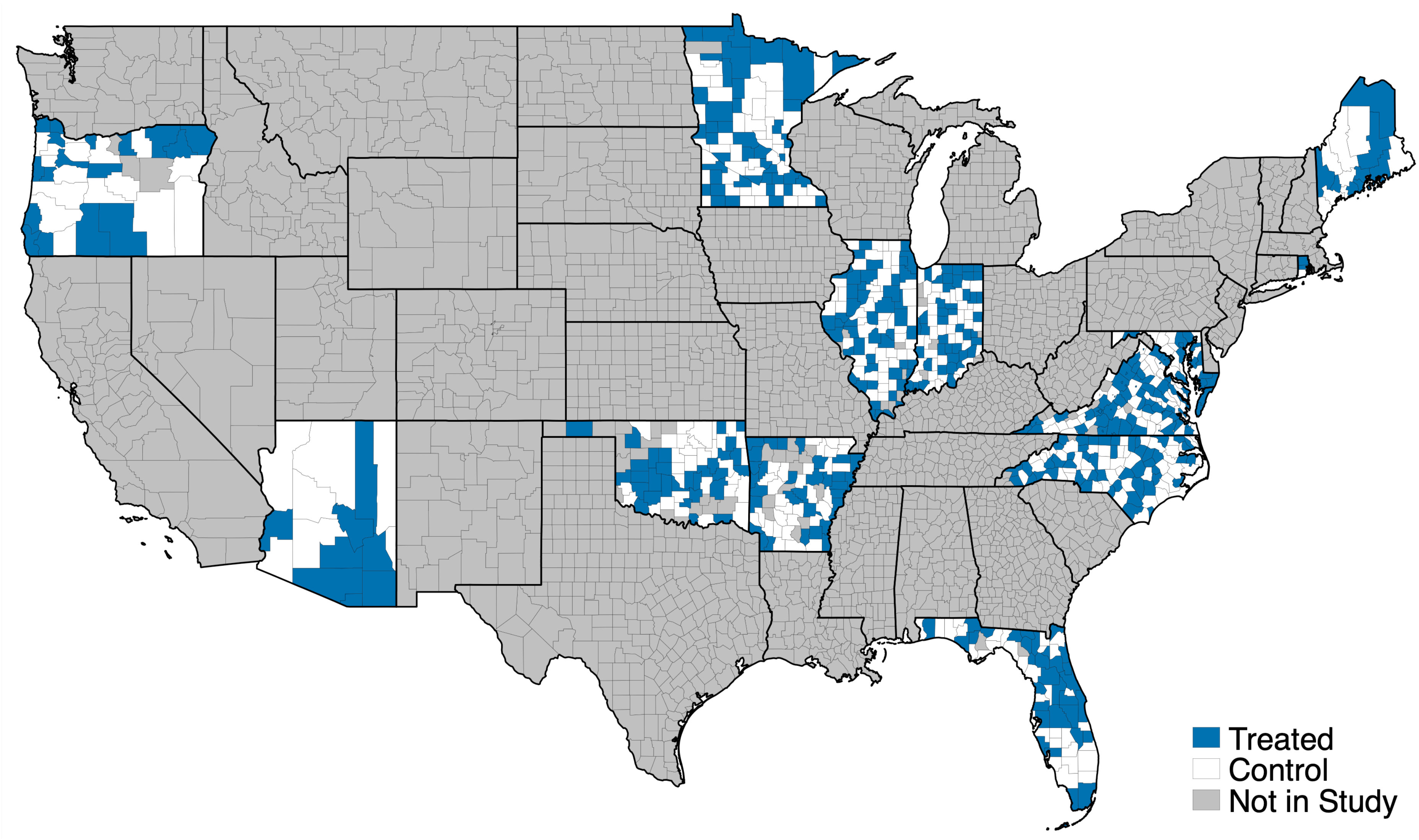
Treated
Control
Not in Study

Figure S2. Day by day difference between high and low intensity counties on Share Ever Left Home*

PANEL A: Thanksgiving Campaign

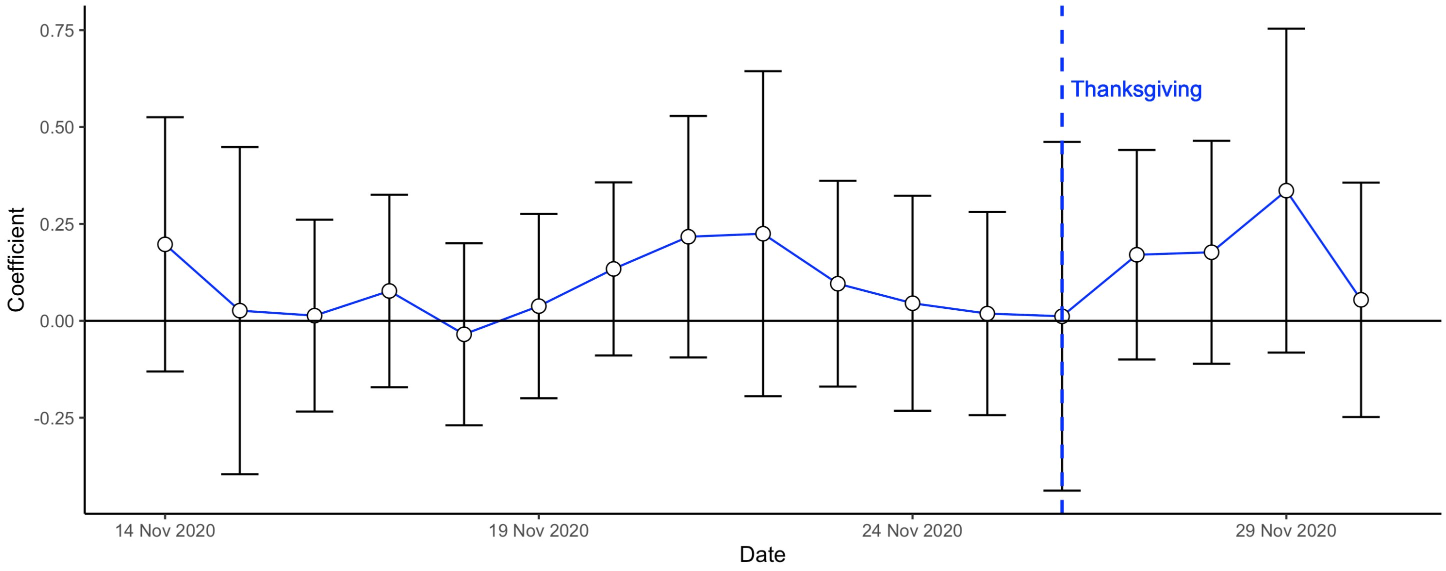

PANEL B: Christmas Campaign

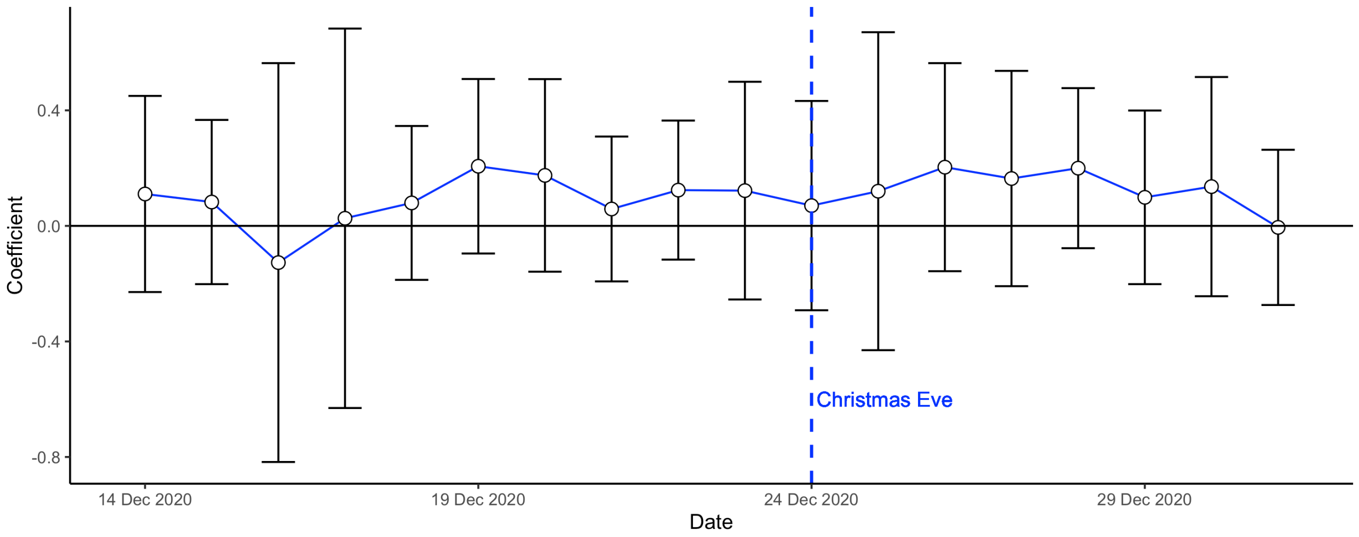

*These Figures show a day by day estimation of the regression equation (1). The outcome is Share Ever Left Home.

Table S2a. Analyses of Mobility Outcomes by Baseline Covid-19 Cases*

			OLS model						Number of days * counties
**Campaign**	**Outcome**	**Period**	**High county High baseline**	**p-value**	**High county**	**p-value**	**High baseline**	**p-value**	
**Both campaigns**	**Distance Traveled**	**from d-3 to d-1**	0.811 (-0.579,2.202)	0.253	-1.484 (-2.736,-0.231)	0.020	-0.518 (-1.727,0.690)	0.401	4059
	**Share Ever Left Home**	**Thanksgiving (Nov 26)/ Christmas (Dec 24-25)**	-0.471 (-1.309,0.368)	0.271	0.325 (-0.380,1.029)	0.367	0.695 (0.128,1.263)	0.016	2017
**Thanksgiving**	**Distance Traveled**	**from d-3 to d-1**	1.509 (-0.369,3.387)	0.115	-1.813 (-3.479,-0.146)	0.033	-0.744 (-2.210,0.722)	0.320	2072
	**Share Ever Left Home**	**Thanksgiving (Nov 26)**	0.082 (-0.889,1.053)	0.869	-0.052 (-0.889,0.784)	0.903	0.404 (-0.255,1.064)	0.230	689
**Christmas**	**Distance Traveled**	**from d-3 to d-1**	0.738 (-1.110,2.586)	0.434	-1.518 (-3.179,0.142)	0.073	-0.853 (-2.297,0.590)	0.247	1987
	**Share Ever Left Home**	**Christmas (Dec 24-25)**	-0.123 (-0.993,0.746)	0.781	0.181 (-0.580,0.943)	0.640	0.300 (-0.323,0.922)	0.345	1328

*This Table gives the control and treatment means at the county level and different periods, in addition to estimates of equation (1) coefficients (here, an interaction with High Baseline is added to the equation) . Standard errors are clustered at the county level. 95% CI are reported in parentheses. High Baseline is defined as: Cumulative Covid-19 county cases per capita at baseline above median.

Table S2b. Analyses of Covid-19 Outcome by Baseline Covid-19 Cases *

Campaign	Outcome	Period	County treatment	OLS model						Number of zip codes
				Treated x High baseline	p-value	Treated	p-value	High baseline	p-value	
Both campaigns	Asinh(Fortnightly Cases)	dec/jan 01-14	All	0.047 (-0.003,0.096)	0.065	-0.058 (-0.102,-0.014)	0.009	0.311 (0.259,0.363)	0.000	13489
			Low Intensity	0.059 (-0.015,0.133)	0.119	-0.061 (-0.122,0.001)	0.054	0.165 (0.105,0.225)	0.000	6723
			High Intensity	0.047 (-0.027,0.121)	0.214	-0.064 (-0.127,-0.001)	0.048	0.240 (0.159,0.321)	0.000	6766
Thanksgiving	Asinh(Fortnightly Cases)	dec 01-14	All	0.039 (-0.022,0.101)	0.208	-0.047 (-0.100,0.006)	0.082	0.095 (0.031,0.159)	0.004	6773
			Low Intensity	0.035 (-0.065,0.135)	0.496	-0.033 (-0.115,0.049)	0.434	0.075 (-0.010,0.159)	0.082	3294
			High Intensity	0.048 (-0.042,0.139)	0.292	-0.064 (-0.141,0.013)	0.105	0.107 (0.006,0.209)	0.038	3479
Christmas	Asinh(Fortnightly Cases)	jan 01-14	All	0.060 (0.004,0.115)	0.035	-0.073 (-0.123,-0.022)	0.005	0.011 (-0.046,0.067)	0.714	6716
			Low Intensity	0.082 (-0.006,0.169)	0.067	-0.091 (-0.166,-0.015)	0.018	-0.049 (-0.119,0.022)	0.178	3429
			High Intensity	0.020 (-0.069,0.110)	0.654	-0.047 (-0.126,0.032)	0.241	0.095 (-0.003,0.194)	0.058	3287

*This Table gives the control and treatment means at the zip level, in addition to the estimate of the treatment coefficient in equation (2). An interaction with High Covid-19 Baseline was added to the equation. The outcome is the log of the Fortnightly Cases, during a period which starts 5 to 7 days after the event (Thanksgiving or Christmas). 95% CI are reported in parentheses. High Baseline is defined as: Cumulative Covid-19 zip cases at baseline above median.

Table S3a. Analyses of Mobility Outcomes by Party Majority*

Campaign	Outcome	Period	OLS model: High county x Majority Rep	p-value	High county	p-value	Majority Rep	p-value	Number of days*counties
Both campaigns	Distance Traveled	from d-3 to d-1	-0.949 (-2.172,0.274)	0.128	-0.240 (-1.211,0.731)	0.628	0.881 (-0.267,2.030)	0.133	4059
	Share Ever Left Home	Thanksgiving (Nov 26)/ Christmas (Dec 24-25)	0.024 (-0.939,0.988)	0.960	0.011 (-0.848,0.869)	0.981	0.009 (-0.606,0.624)	0.977	2017
Thanksgiving	Distance Traveled	from d-3 to d-1	-0.632 (-2.545,1.282)	0.518	-0.422 (-2.067,1.223)	0.615	0.448 (-1.087,1.983)	0.567	2072
	Share Ever Left Home	Thanksgiving (Nov 26)	0.085 (-1.096,1.265)	0.888	-0.056 (-1.125,1.013)	0.918	-0.143 (-0.920,0.635)	0.719	689
Christmas	Distance Traveled	from d-3 to d-1	-1.472 (-3.208,0.264)	0.097	0.122 (-1.340,1.585)	0.870	1.475 (0.240,2.711)	0.019	1987
	Share Ever Left Home	Christmas (Dec 24-25)	-0.245 (-1.156,0.666)	0.598	0.287 (-0.513,1.087)	0.482	0.280 (-0.352,0.911)	0.385	1328

*This Table gives the control and treatment means at the county level and different periods, in addition to estimates of equation (1) coefficients (here, an interaction with Republican Majority is added to the equation) . Standard errors are clustered at the county level. 95% CI are reported in parentheses. Republican Majority is defined by “share of republican voters > share of democrat voters” in the county.

Table S3b. Analyses of Covid Outcome by Party Majority*

Campaign	Outcome	Period	County treatment	OLS model: Treated x Majority Rep	p-value	Treated	p-value	Number of zip codes
Both campaigns	Asinh(Fortnightly Cases)	dec/jan 01-14	All	-0.001 (-0.052,0.050)	0.975	-0.034 (-0.073,0.005)	0.087	13489

			Low Intensity	-0.044 (-0.112,0.024)	0.209	-0.003 (-0.051,0.045)	0.901	6723
			High Intensity	0.001 (-0.071,0.073)	0.979	-0.040 (-0.095,0.015)	0.156	6766
Thanksgiving	Asinh(Fortnightly Cases)	dec 01-14	All	-0.046 (-0.111,0.019)	0.164	0.004 (-0.047,0.054)	0.886	6773
			Low Intensity	-0.046 (-0.144,0.053)	0.360	0.016 (-0.062,0.094)	0.692	3294
			High Intensity	-0.047 (-0.132,0.039)	0.286	-0.008 (-0.073,0.057)	0.817	3479
Christmas	Asinh(Fortnightly Cases)	jan 01-14	All	-0.017 (-0.077,0.043)	0.572	-0.031 (-0.076,0.014)	0.175	6716
			Low Intensity	-0.063 (-0.143,0.017)	0.123	-0.008 (-0.063,0.047)	0.780	3429
			High Intensity	0.032 (-0.059,0.123)	0.491	-0.057 (-0.130,0.015)	0.122	3287

*This Table gives the control and treatment means at the zip level, in addition to the estimate of the treatment coefficient in equation (2). An interaction with Republican Majority was added to the equation. The outcome is the Inverse Hyperbolic Sine of the Fortnightly Cases, during a period which starts 5 to 7 days after the event (Thanksgiving or Christmas). 95% CI are reported in parentheses. Republican Majority is defined by “share of republican voters > share of democrat voters” in the county.

Table S3c. Analyses of Mobility Outcomes: Urban vs Rural*

			OLS model						Number of days*counties
Campaign	Outcome	Period	High county x Majority urban	p-value	High county	p-value	Majority urban	p-value	
Both campaigns	Distance Traveled	from d-3 to d-1	0.089 (-1.130,1.309)	0.886	-1.025 (-1.920,-0.130)	0.025	-0.497 (-1.512,0.517)	0.337	4056
	Share Ever Left Home	Thanksgiving (Nov 26)/ Christmas (Dec 24-25)	-0.385 (-1.157,0.386)	0.327	0.203 (-0.343,0.750)	0.466	-0.089 (-0.599,0.421)	0.733	2015
Thanksgiving	Distance Traveled	from d-3 to d-1	0.270 (-1.380,1.919)	0.749	-1.027 (-2.302,0.249)	0.115	-0.502 (-1.769,0.765)	0.438	2072
	Share Ever Left Home	Thanksgiving (Nov 26)	-0.521 (-1.401,0.359)	0.246	0.233 (-0.404,0.870)	0.474	0.197 (-0.414,0.808)	0.527	689
Christmas	Distance Traveled	from d-3 to d-1	0.074 (-1.473,1.621)	0.925	-1.077 (-2.310,0.156)	0.087	-0.701 (-1.852,0.451)	0.233	1984

	Share Ever Left Home	Christmas (Dec 24-25)	-0.205 (-0.947,0.538)	0.589	0.184 (-0.385,0.753)	0.526	-0.442 (-0.972,0.087)	0.102	1326

*This Table gives the control and treatment means at the county level and different periods, in addition to estimates of equation (1) coefficients (here, an interaction with Urban Majority is added to the equation) . Standard errors are clustered at the county level. 95% CI are reported in parentheses. Urban Majority is defined by a majority of urban zip codes in the county.

Table S3d. Analyses of Covid Outcome: Urban vs Rural *

				OLS model				Number of zip codes
Campaign	Outcome	Period	County treatment	Treated x Majority urban	p-value	Treated	p-value	
Both campaigns	Asinh(Fortnightly Cases)	dec/jan 01-14	All	0.037 (-0.016,0.090)	0.176	-0.054 (-0.100,-0.008)	0.021	13489
			Low Intensity	0.059 (-0.014,0.132)	0.114	-0.063 (-0.127,0.001)	0.053	6723
			High Intensity	0.020 (-0.053,0.092)	0.597	-0.049 (-0.110,0.012)	0.115	6766
Thanksgiving	Asinh(Fortnightly Cases)	dec 01-14	All	0.046 (-0.019,0.111)	0.163	-0.051 (-0.104,0.003)	0.062	6773
			Low Intensity	0.051 (-0.044,0.146)	0.294	-0.041 (-0.117,0.036)	0.300	3294
			High Intensity	0.043 (-0.045,0.130)	0.339	-0.061 (-0.135,0.013)	0.105	3479
Christmas	Asinh (Fortnightly Cases)	jan 01-14	All	0.030 (-0.034,0.093)	0.358	-0.058 (-0.113,-0.004)	0.037	6716
			Low Intensity	0.054 (-0.037,0.145)	0.246	-0.079 (-0.160,0.003)	0.059	3429
			High Intensity	0.006 (-0.083,0.094)	0.900	-0.039 (-0.112,0.034)	0.297	3287

*This Table gives the control and treatment means at the zip level, in addition to the estimate of the treatment coefficient in equation (2). An interaction with Urban Majority was added to the equation. The outcome is the Inverse Hyperbolic Sine of the Fortnightly

Cases, during a period which starts 5 to 7 days after the event (Thanksgiving or Christmas). 95% CI are reported in parentheses. Urban Majority is defined by a majority of urban zip codes in the county.

Table S3e Analyses of Mobility Outcomes by Republican Majority x Urban Majority*

			OLS model								Number of days*counties
Campaign	Outcome	Period	High x Majority urban x Majority rep	p-value	High x Majority rep	p-value	High x Majority urban	p-value	High county	p-value	
Both campaigns	Distance Traveled	from d-3 to d-1	0.378 (-2.464,3.219)	0.794	-1.199 (-3.598,1.200)	0.327	-0.446 (-2.864,1.971)	0.718	0.036 (-2.158,2.231)	0.974	4056
	Share Ever Left Home	Thanksgiving (Nov 26)/ Christmas (Dec 24-25)	-0.932 (-3.192,1.328)	0.419	0.490 (-1.455,2.435)	0.621	0.341 (-1.741,2.424)	0.748	-0.231 (-2.092,1.630)	0.808	2015
Thanksgiving	Distance Traveled	from d-3 to d-1	-0.848 (-5.709,4.014)	0.733	-0.069 (-4.485,4.347)	0.976	0.814 (-3.650,5.277)	0.721	-0.964 (-5.184,3.255)	0.654	2072
	Share Ever Left Home	Thanksgiving (Nov 26)	-0.382 (-3.251,2.486)	0.794	0.153 (-2.409,2.715)	0.907	-0.242 (-2.937,2.453)	0.860	0.097 (-2.377,2.572)	0.938	689
Christmas	Distance Traveled	from d-3 to d-1	0.999 (-3.534,5.533)	0.666	-2.110 (-6.294,2.074)	0.323	-1.111 (-5.264,3.042)	0.600	0.793 (-3.188,4.773)	0.696	1984
	Share Ever Left Home	Christmas (Dec 24-25)	-1.962 (-4.245,0.321)	0.092	0.957 (-1.106,3.021)	0.363	1.305 (-0.815,3.426)	0.227	-0.667 (-2.646,1.311)	0.508	1326

*This Table gives the control and treatment means at the county level and different periods, in addition to estimates of equation (1) coefficients (here, an interaction with Urban Majority and Republican Majority is added to the equation) . Standard errors are clustered at the county level. 95% CI are reported in parentheses. Urban Majority is defined by a majority of urban zip codes in the county. Republican Majority is defined by “share of republican voters > share of democrat voters” in the county.

Table S3f. Analyses of Covid Outcome by Republican Majority x Urban Majority*

				OLS model								Number of zip codes
Campaign	Outcome	Period	County treatment	Treated x Majority urban x Majority rep	p-value	Treated x Majority rep	p-value	Treated x Majority urban	p-value	Treated	p-value	
Both campaigns	Asinh(Fortnightly Cases)	dec/jan 01-14	All	-0.129 (-0.278,0.021)	0.092	0.113 (-0.026,0.251)	0.112	0.143 (0.008,0.278)	0.038	-0.153 (-0.283,-0.023)	0.021	13489
			Low Intensity	-0.003 (-0.215,0.208)	0.975	-0.017 (-0.216,0.182)	0.869	0.053 (-0.139,0.246)	0.587	-0.048 (-0.235,0.139)	0.614	6723
			High Intensity	-0.135 (-0.348,0.078)	0.215	0.109 (-0.089,0.308)	0.279	0.128 (-0.067,0.323)	0.198	-0.146 (-0.333,0.042)	0.128	6766
Thanksgiving	Asinh(Fortnightly Cases)	dec 01-14	All	0.110 (-0.080,0.301)	0.255	-0.110 (-0.286,0.066)	0.220	-0.052 (-0.226,0.122)	0.561	0.047 (-0.120,0.213)	0.583	6773
			Low Intensity	0.118 (-0.154,0.389)	0.396	-0.109 (-0.356,0.138)	0.386	-0.048 (-0.295,0.198)	0.700	0.055 (-0.178,0.288)	0.644	3294
			High Intensity	0.103 (-0.164,0.369)	0.451	-0.109 (-0.358,0.140)	0.390	-0.052 (-0.297,0.193)	0.677	0.037 (-0.200,0.274)	0.761	3479
Christmas	Asinh(Fortnightly Cases)	jan 01-14	All	-0.220 (-0.411,-0.030)	0.023	0.157 (-0.022,0.337)	0.086	0.197 (0.021,0.372)	0.028	-0.197 (-0.367,-0.027)	0.023	6716
			Low Intensity	-0.084 (-0.401,0.232)	0.602	0.018 (-0.287,0.324)	0.906	0.099 (-0.198,0.397)	0.513	-0.095 (-0.389,0.198)	0.525	3429
			High Intensity	-0.322 (-0.562,-0.082)	0.009	0.263 (0.044,0.482)	0.019	0.260 (0.042,0.478)	0.019	-0.265 (-0.470,-0.061)	0.011	3287

*This Table gives the control and treatment means at the zip level, in addition to the estimate of the treatment coefficient in equation (2). An interaction with Urban Majority and Republican Majority was added to the equation. The outcome is the log of the Fortnightly Cases, during a period which starts 5 to 7 days after the event (Thanksgiving or Christmas). 95% CI are reported in parentheses. Urban Majority is defined by a majority of urban zip codes in the county. Republican Majority is defined by “share of republican voters > share of democrat voters” in the county.

Table S3e. Analyses of Mobility Outcomes by Education*

OLS model	

Campaign	Outcome	Period	High county x High educ	p-value	High county	p-value	High educ	p-value	Number of days*counties
Both campaigns	Distance Traveled	from d-3 to d-1	-0.329 (-1.591,0.932)	0.609	-0.835 (-1.562,-0.108)	0.024	0.293 (-0.782,1.368)	0.593	4059
	Share Ever Left Home	Thanksgiving (Nov 26)/ Christmas (Dec 24-25)	0.380 (-0.402,1.161)	0.341	-0.146 (-0.667,0.375)	0.583	0.215 (-0.306,0.736)	0.419	2017
Thanksgiving	Distance Traveled	from d-3 to d-1	-0.147 (-1.889,1.595)	0.869	-0.845 (-1.797,0.107)	0.082	0.255 (-1.080,1.589)	0.708	2072
	Share Ever Left Home	Thanksgiving (Nov 26)	0.057 (-0.840,0.954)	0.901	-0.001 (-0.590,0.589)	0.998	0.402 (-0.220,1.024)	0.205	689
Christmas	Distance Traveled	from d-3 to d-1	-0.893 (-2.530,0.744)	0.285	-0.625 (-1.600,0.349)	0.208	0.632 (-0.625,1.889)	0.325	1987
	Share Ever Left Home	Christmas (Dec 24-25)	0.252 (-0.518,1.023)	0.521	-0.026 (-0.525,0.473)	0.918	0.390 (-0.159,0.938)	0.164	1328

*This Table gives the control and treatment means at the county level and different periods, in addition to estimates of equation (1) coefficients (here, an interaction with High Education is added to the equation) . Standard errors are clustered at the county level. 95% CI are reported in parentheses. High Education is defined by a proportion of high school graduates (aged > 25) in county above median.

Table S3g. Analyses of Covid Outcome by Education*

				OLS model				Number of zip codes
Campaign	Outcome	Period	County treatment	Treated x High educ	p-value	Treated	p-value	
Both campaigns	Asinh(Fortnightly Cases)	dec/jan 01-14	All	0.002 (-0.054,0.059)	0.941	-0.036 (-0.067,-0.004)	0.027	13489
			Low Intensity	0.004 (-0.077,0.086)	0.919	-0.033 (-0.072,0.006)	0.096	6723
			High Intensity	-0.028 (-0.106,0.049)	0.476	-0.028 (-0.070,0.013)	0.184	6766
Thanksgiving	Asinh(Fortnightly Cases)	dec 01-14	All	-0.039 (-0.108,0.031)	0.276	-0.012 (-0.049,0.025)	0.510	6773
			Low Intensity	0.018 (-0.084,0.120)	0.729	-0.022 (-0.078,0.034)	0.440	3294

			High Intensity	-0.094 (-0.189,0.000)	0.050	-0.004 (-0.052,0.045)	0.883	3479
Christmas	Asinh(Fortnightly Cases)	jan 01-14	All	-0.001 (-0.067,0.065)	0.984	-0.042 (-0.078,-0.006)	0.023	6716
			Low Intensity	-0.033 (-0.130,0.064)	0.502	-0.038 (-0.087,0.010)	0.122	3429
			High Intensity	0.026 (-0.065,0.118)	0.573	-0.047 (-0.100,0.007)	0.091	3287

*This Table gives the control and treatment means at the zip level, in addition to the estimate of the treatment coefficient in equation (2). An interaction with High Education was added to the equation. The outcome is the Inverse Hyperbolic Sine of the Fortnightly Cases, during a period which starts 5 to 7 days after the event (Thanksgiving or Christmas). 95% CI are reported in parentheses. High Education is defined by a proportion of high school graduates (aged > 25) in county above median.

Table S4. Effect of Intervention on Movement Outcomes, with Double Post Lasso Control Variables*

			Mean (95% CI)		OLS model		Number of days * counties
Campaign	Outcome	Period	High county	Low county	High county coef (95% CI)	p-value	
Both campaigns	Distance Traveled	from d-3 to d-1	-4.384 (-4.973,-3.796)	-3.603 (-4.254,-2.952)	-0.950 (-1.558,-0.342)	0.002	4059
	Share Ever Left Home	Thanksgiving (Nov 26)/ Christmas (Dec 24-25)	72.326 (72.012,72.639)	72.381 (72.092,72.670)	-0.008 (-0.380,0.364)	0.968	2017
Thanksgiving	Distance Traveled	from d-3 to d-1	-6.082 (-6.822,-5.341)	-5.320 (-6.113,-4.527)	-0.731 (-1.528,0.067)	0.073	2072
	Share Ever Left Home	Thanksgiving (Nov 26)	71.308 (70.885,71.731)	71.468 (71.071,71.866)	0.074 (-0.258,0.406)	0.662	689
Christmas	Distance Traveled	from d-3 to d-1	-2.603 (-3.279,-1.927)	-1.823 (-2.588,-1.057)	-1.004 (-1.764,-0.244)	0.010	1987
	Share Ever Left Home	Christmas (Dec 24-25)	72.859 (72.507,73.210)	72.852 (72.520,73.185)	0.074 (-0.235,0.384)	0.638	1328

*This Table gives the control and treatment means at the county level and different periods, in addition to the estimate of the treatment coefficient in equation (1). Controls (county covariates and state fixed effects) are selected via Double Post Lasso. Standard errors are clustered at the county level. 95% CI are reported in parentheses.

Table S5. Effect of Intervention on Covid-19 Outcome: median regression*

				Median regression		Number of zip codes
Campaign	Outcome	Period	County treatment	coef (CI 95%)	p-value	
Both campaigns	log(Fortnightly Cases+1)	dec/jan 01-14	All	-0.020 (-0.039,-0.001)	0.037	13489
			Low Intensity	0.004 (-0.020,0.027)	0.745	6723
			High Intensity	-0.031 (-0.053,-0.010)	0.004	6766
Thanksgiving	log(Fortnightly Cases+1)	dec 01-14	All	-0.004 (-0.026,0.017)	0.694	6773
			Low Intensity	0.010 (-0.027,0.046)	0.605	3294
			High Intensity	-0.015 (-0.049,0.020)	0.404	3479
Christmas	log(Fortnightly Cases+1)	jan 01-14	All	-0.021 (-0.043,0.001)	0.061	6716
			Low Intensity	-0.006 (-0.039,0.027)	0.716	3429
			High Intensity	-0.033 (-0.066,0.000)	0.049	3287

*This Table gives the median treatment effects on Covid-19 cases at the zip level. The outcome is log(Fortnightly Cases +1), during a period which starts 5 to 7 days after the event (Thanksgiving or Christmas). 95% CI are reported in parentheses. The coefficients were estimated with the Barrodale and Roberts algorithm (quantreg R package). Standard errors were obtained with the bootstrap method.

Table S6a. Effect of Intervention on Covid-19 Outcome (both campaigns), robustness to function form

				Mean (CI 95%)		OLS model		Number of zip codes
**Specification**	**Outcome**	**Period**	**County treatment**	**Treatment**	**Control**	**Treatment (CI 95%)**	**p-value**	
Fortnightly cases zeros are omitted	**Log(Fortnightly Cases)**	**dec/jan 01-14**	**All**	3.718 (3.672,3.764)	3.745 (3.700,3.790)	-0.033 (-0.060,-0.007)	0.013	13269
			**Low Intensity**	3.733 (3.649,3.816)	3.738 (3.687,3.788)	-0.036 (-0.070,-0.001)	0.042	6603
			**High Intensity**	3.713 (3.663,3.764)	3.767 (3.688,3.847)	-0.034 (-0.069,0.000)	0.051	6666
Fortnightly cases zeros are replaced with min(positive Fortnightly cases)/2	**Log(Fortnightly Cases)**	**dec/jan 01-14**	**All**	3.649 (3.601,3.697)	3.670 (3.623,3.717)	-0.036 (-0.064,-0.008)	0.011	13489
			**Low Intensity**	3.657 (3.570,3.744)	3.657 (3.604,3.711)	-0.034 (-0.070,0.002)	0.066	6723
			**High Intensity**	3.646 (3.593,3.699)	3.707 (3.624,3.790)	-0.040 (-0.076,-0.003)	0.033	6766
Adding 1	**Log(Fortnightly Cases+1)**	**dec/jan 01-14**	**All**	3.732 (3.687,3.777)	3.750 (3.706,3.794)	-0.030 (-0.054,-0.005)	0.020	13489
			**Low Intensity**	3.745 (3.664,3.826)	3.739 (3.689,3.788)	-0.025 (-0.057,0.007)	0.128	6723
			**High Intensity**	3.728 (3.679,3.777)	3.784 (3.707,3.861)	-0.035 (-0.068,-0.003)	0.033	6766

*This Table gives the control and treatment means at the zip level, in addition to the estimate of the treatment coefficient in equation (2). The outcome is a function of the Fortnightly Cases, during a period which starts 5 to 7 days after the event (Thanksgiving or Christmas). 95% CI are reported in parentheses. Standard errors are clustered at the zip level.

Table S6b. Effect of Intervention on Covid-19 Outcome (Thanksgiving campaign), robustness to functional form

				Mean (CI 95%)		OLS model		Number of zip codes
**Specification**	**Outcome**	**Period**	**County treatment**	**Treatment**	**Control**	**Treatment (CI 95%)**	**p-value**	
Fortnightly cases zeros are omitted	**Log(Fortnightly Cases)**	**dec/jan 01-14**	**All**	3.700 (3.646,3.753)	3.660 (3.607,3.713)	-0.022 (-0.053,0.010)	0.172	6672
			**Low Intensity**	3.651 (3.540,3.762)	3.628 (3.567,3.690)	-0.025 (-0.072,0.021)	0.288	3239
			**High Intensity**	3.715 (3.654,3.776)	3.748 (3.644,3.853)	-0.019 (-0.061,0.024)	0.383	3433
Fortnightly cases zeros are replaced with	**Log(Fortnightly Cases)**	**dec/jan 01-14**	**All**	3.632 (3.576,3.687)	3.597 (3.542,3.652)	-0.028 (-0.061,0.004)	0.089	6773
			**Low Intensity**	3.582 (3.466,3.698)	3.555 (3.490,3.619)	-0.017 (-0.066,0.032)	0.495	3294

min(positive Fortnightly cases)/2			**High Intensity**	3.647 (3.584,3.711)	3.718 (3.612,3.824)	-0.039 (-0.083,0.005)	0.079	3479
Adding 1	**Log(Fortnightly Cases+1)**	**dec/jan 01-14**	**All**	3.714 (3.663,3.766)	3.679 (3.627,3.730)	-0.021 (-0.050,0.007)	0.145	6773
			**Low Intensity**	3.670 (3.563,3.778)	3.639 (3.580,3.699)	-0.010 (-0.053,0.032)	0.635	3294
			**High Intensity**	3.728 (3.669,3.787)	3.791 (3.691,3.890)	-0.032 (-0.071,0.007)	0.108	3479

*This Table gives the control and treatment means at the zip level, in addition to the estimate of the treatment coefficient in equation (2). The outcome is a function of the Fortnightly Cases, during a period which starts 5 to 7 days after the event (Thanksgiving). 95% CI are reported in parentheses. Standard errors are clustered at the zip level.

Table S6c. Effect of Intervention on Covid-19 Outcome (Christmas campaign), robustness to functional form

				**Mean (CI 95%)**		**OLS model**		**Number of zip codes**
**Specification**	**Outcome**	**Period**	**County treatment**	**Treatment**	**Control**	**Treatment (CI 95%)**	**p-value**	
Fortnightly cases zeros are omitted	**Log(Fortnightly Cases)**	**dec/jan 01-14**	**All**	3.737 (3.681,3.793)	3.830 (3.775,3.884)	-0.049 (-0.078,-0.020)	0.001	6597
			**Low Intensity**	3.810 (3.696,3.924)	3.844 (3.781,3.906)	-0.050 (-0.090,-0.010)	0.015	3364
			**High Intensity**	3.711 (3.647,3.775)	3.787 (3.678,3.896)	-0.049 (-0.090,-0.007)	0.021	3233
Fortnightly cases zeros are replaced with min(positive Fortnightly cases)/2	**Log(Fortnightly Cases)**	**dec/jan 01-14**	**All**	3.666 (3.608,3.724)	3.742 (3.684,3.799)	-0.044 (-0.075,-0.013)	0.006	6716
			**Low Intensity**	3.727 (3.608,3.846)	3.757 (3.691,3.823)	-0.051 (-0.094,-0.008)	0.021	3429
			**High Intensity**	3.645 (3.578,3.711)	3.695 (3.580,3.811)	-0.037 (-0.082,0.008)	0.109	3287
Adding 1	**Log(Fortnightly Cases+1)**	**dec/jan 01-14**	**All**	3.750 (3.696,3.804)	3.821 (3.767,3.874)	-0.038 (-0.064,-0.011)	0.006	6716
			**Low Intensity**	3.815 (3.704,3.925)	3.835 (3.773,3.897)	-0.039 (-0.076,-0.002)	0.041	3429
			**High Intensity**	3.728 (3.666,3.789)	3.777 (3.670,3.884)	-0.036 (-0.075,0.002)	0.065	3287

*This Table gives the control and treatment means at the zip level, in addition to the estimate of the treatment coefficient in equation (2). The outcome is a function of the Fortnightly Cases, during a period which starts 5 to 7 days after the event (Christmas). 95% CI are reported in parentheses. Standard errors are clustered at the zip level.

Section E. References

2. Chernozhukov V, Chetverikov D, Demirer M, et al. Double/debiased machine learning for treatment and structural parameters. Econom J 2018;21(1):C1-C68.

Supplement 2. Statistical Analysis Plan

The Statistical Analysis Plan can be accessed via this link: https://www.dropbox.com/s/ctqdw24vy2g3haq/NEJM_Statistical_Analysis_Plan.pdf?dl=0.
